# The effect of individualized breastfeeding support on exclusive breastfeeding in the first 6 months, occurrence of breast problems and breastfeeding self-efficacy: randomized controlled study

**DOI:** 10.1186/s12884-026-09274-z

**Published:** 2026-05-21

**Authors:** Seçil Köken Durgun, Seval Cambaz Ulaş

**Affiliations:** Department of Midwifery, Faculty of Health Sciences, Manisa Celal Bayar University, 45120 Manisa, Türkiye

**Keywords:** Lactation management, Exclusive breast milk, Breast problems, Breastfeeding self-efficacy, Breastfeeding success

## Abstract

**Background:**

This study aimed to determine the effect of simulator-assisted individualized breastfeeding support given to mothers on their breastfeeding exclusively for the first six months, breastfeeding problems, and breastfeeding competencies.

**Methods:**

This was a randomized controlled study. The population of the study consisted of pregnant women who applied to hospital in the west of Türkiye (*N* = 1800). 102 pregnant women were included in study (intervention = 51, control = 51; *n* = 102). Pregnant women in the intervention group received simulator-assisted breastfeeding education and a booklet explaining the breastfeeding process during the antenatal period, while the control group received only a booklet. Exclusive breastfeeding of infants in the first six months postpartum, occurrence of breast problems, and breastfeeding competencies of mothers were monitored by home visits on the fifth day, the fifteenth day, and the first month of the postpartum period and by telephone in the third and sixth months.

**Results:**

The rate of exclusive breastfeeding was significantly higher in the intervention group compared to the control group in all follow-ups. Breast problems were observed less frequently in the mothers of the intervention group in all follow-ups. Breastfeeding self-efficacy scale scores were also found to be significantly higher in the intervention group mothers compared to the control group.

**Conclusions:**

As a result of the research, it was determined that mothers who received individual breastfeeding counseling fed their babies exclusively with breast milk for a longer period of time, had fewer breast problems, and had higher breastfeeding adequacy.

**Trial registration:**

Study findings were recorded in the Clinical Trial System (Registration No: NCT06341140, Registration Date: 02.04.2024).

## Introduction

Breastfeeding is the most ideal method for infant nutrition. Breast milk, which is secreted uniquely for each baby, consists of content that will ensure growth and development [1]. The World Health Organization (WHO) and the United Nations Children’s Fund (UNICEF) recommend starting breastfeeding within the first hour after birth at the latest. For the first six months, they support exclusive breastfeeding without any other food, including water. It recommends continuing breastfeeding until the age of two years in addition to complementary feeding after the sixth month [2, 3]. Despite efforts to promote and support breastfeeding, global breastfeeding indicators show that breastfeeding rates are below targeted levels [4]. According to Global Breastfeeding Scorecard by UNICEF, WHO, and Global Breastfeeding Collective 2023 report, 46% of babies start breastfeeding within the first hour after birth, and 48% of babies under six months of age are exclusively breastfed. Between 12 and 23 months, 45% of infants continue to receive breast milk [5]. In Türkiye, the rate of breastfeeding within the first hour after birth is 71.3%, exclusive breastfeeding is 40.7% in infants under six months of age, and breastfeeding up to two years of age is 33.3% [6].

In a breastfeeding-friendly environment, the vast majority of women are biologically able to breastfeed successfully. Few medical conditions prevent breastfeeding [7, 8]. According to the research findings, while breastfeeding is widespread, challenges such as non-exclusive breastfeeding during the first six months, premature cessation of breastfeeding, and early introduction of supplementary foods are commonly encountered. The emergence of these problems can be attributed to failure to initiate breastfeeding early after birth, giving food other than breast milk to the newborn, lack of knowledge of the mother, lack of support from health personnel after discharge, slow weight gain of the baby, low self-efficacy of the mother and breast problems of the mother [9, 10]. There are data showing that initiation of breastfeeding is widespread in health institutions in Türkiye because they are baby-friendly hospitals, but the rates of exclusive breastfeeding for the first six months are quite low, and complementary feeding is started early [6]. Babies who are not exclusively breastfed for the first six months have changes in the duration and frequency of breastfeeding. This can directly lead to breast problems. In addition to changes in the baby’s nutrition, breast problems can also develop due to the use of incorrect breastfeeding techniques and positions. A mother with breast problems may experience pain and, therefore, avoid breastfeeding. Therefore, she feels inadequate, and breastfeeding self-efficacy decreases. Consequently, there are interruptions in the continuation of breastfeeding, rates of exclusive breastfeeding in the first six months after birth decrease, and complementary feeding is started earlier than the sixth month [9, 10]. As a result, in the first six months after birth, infant feeding, breast problems, and the mother’s breastfeeding self-efficacy affect each other, and it is seen that it draws a framework that becomes a vicious circle with the mistakes made. It is clear that mothers need continuous individualized breastfeeding support during the initiation, maintenance, and management of breastfeeding. Breastfeeding support should be initiated with breastfeeding training given by a trained health personnel, especially in the antenatal period. In the postpartum period, breastfeeding success will be increased by repeating the information given during pregnancy and correcting incorrect practices, which will positively affect self-efficacy [11]. In this way, breastfeeding will be initiated correctly and successful breastfeeding will be achieved and continued.

Studies on this subject in Türkiye have shown that breastfeeding support is only given before or after birth. At the same time, there are few studies on the sustainability of breastfeeding. In this context, a research project was planned to determine the effect of simulator-assisted individualized breastfeeding education given in antenatal and continued breastfeeding support in postpartum on exclusive breastfeeding for the first 6 months, the development of breast problems, and breastfeeding self-efficacy.

## Materials and methods

### Research type

This is a randomized, controlled interventional study.

### Research location

The study was conducted between August 2021 and November 2022. The initial phase of including pregnant women in the research groups took place at a university hospital in western Türkiye. The hospital holds a Baby-Friendly Hospital certification and operates an active breastfeeding clinic and a childbirth school. Antenatal and postpartum care is generally provided by midwives and nurses. Women who have uncomplicated vaginal deliveries are usually discharged after 24 h, while cesarean deliveries result in a 48-hour hospital stay. Pregnant women admitted to the hospital receive care from the initial antenatal check-up to postpartum follow-up. The postpartum period of the study was carried out through home visits and telephone counseling.

### Sample

**Fig. 1:**
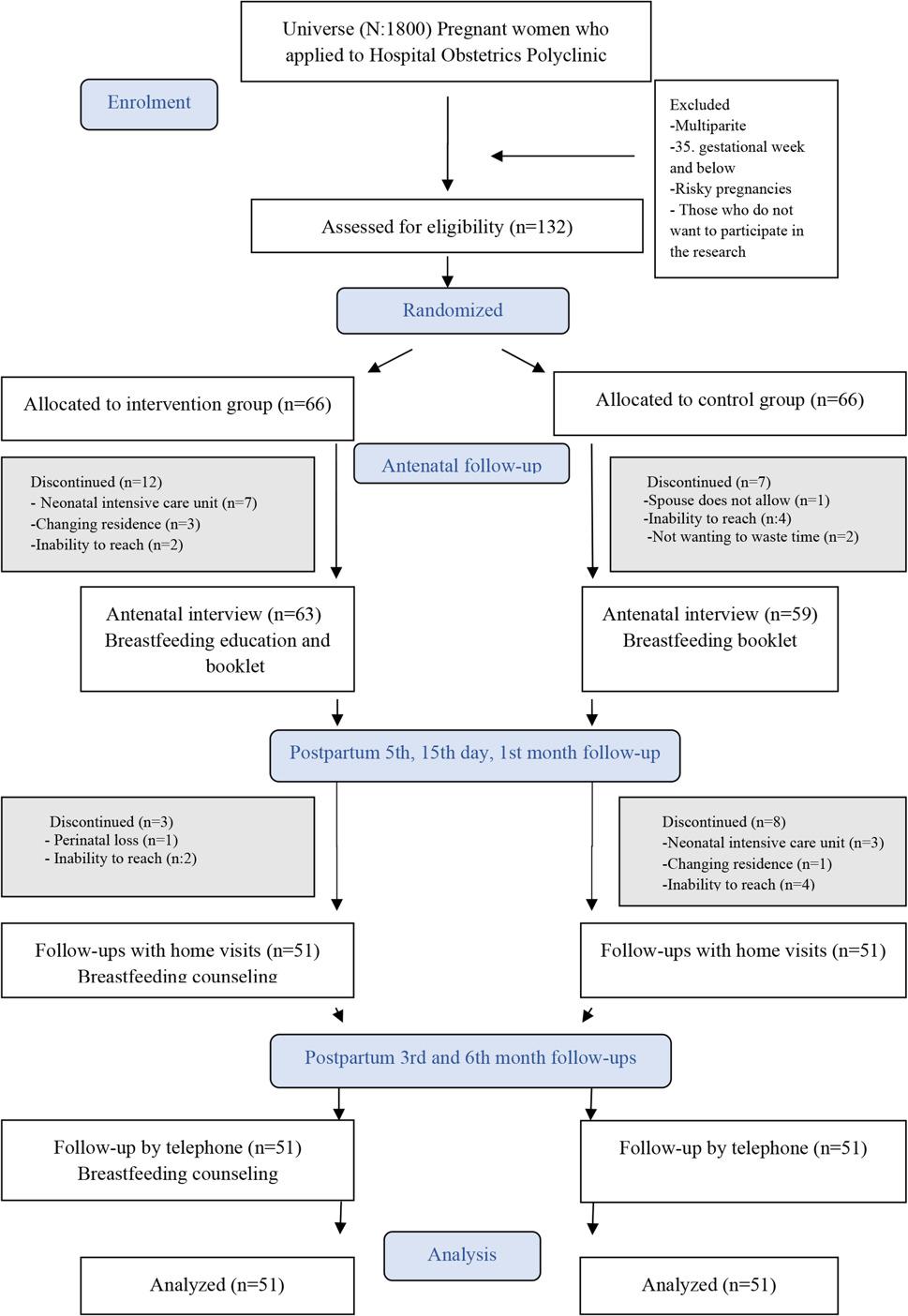
CONSORT 2025 Flow Diagram

The study sample consisted of pregnant women who presented to the obstetrics and gynecology outpatient clinic of a university hospital in Western Türkiye. This number was 1800 for the year 2020 (*N* = 1800). The smallest sample size was determined as 102 (intervention group = 51 and control group = 51) using the LATCH Breastfeeding Diagnostic Tool data of a similar study (intervention group = 9.82 and control group = 8.46) [12], with a margin of error of 0.05, power of 80%, and a moderate effect size (d = 0.50) using the G-power program. In the intervention group, three pregnant women were excluded from the study due to perinatal loss (*n* = 1) and inability to reach the pregnant woman (*n* = 2) before the antenatal interview. After delivery, 12 mothers were excluded from the study because the baby was hospitalized in the neonatal intensive care unit (*n* = 7), the mother could not be reached (*n* = 2), and the mother started to live in another city (*n* = 3). In the control group, seven pregnant women were excluded from the study because the pregnant woman wanted to leave the study before the antenatal interview (*n* = 3), and the pregnant woman could not be reached (*n* = 4). After delivery, eight mothers dropped out of the study because the baby was hospitalized in the neonatal intensive care unit (*n* = 3), the mother could not be reached (*n* = 4), and the mother started to live in another city (*n* = 1). As a result of these losses, the study was completed with 102 pregnant women (intervention = 51 and control = 51) (Fig. 1). Post hoc power analysis was performed after the study, and the power was found to be 0.99.

In this study, a randomization grid obtained from a randomization website (www.random.org) was used to determine which group the pregnant women would be in. Pregnant women who met the inclusion criteria and agreed to participate in the study were assigned to the intervention and control groups by the principal investigator using the randomization table. Participants do not know which group they are in. Three criteria were taken into consideration in the randomization of the intervention and control groups: maternal age, educational status (high school and below/university and above), and employment status (working/not working). It was observed that the groups were homogeneously distributed in terms of the randomization criteria (*p* > 0.05) (Table 1).

**Table 1:** Sociodemographic characteristics of pregnant women in the intervention and control groups

Characteristics		Intervention group		Control group		Significance test
		*n*	%	*n*	%	x^2^ / *p*
Age (year)X̄±Ss(Min/Max)25.44 ± 3.37 (30/35)	20–24	21	41.2	25	49.0	0.748 / 0.68
	25–29	22	43.1	18	35.3	
	30 and above	8	15.7	8	15.7	
Education level	High school and below	23	45.1	26	51.0	0.353 / 0.55
	University and above	28	54.9	25	49.0	
Working status	Yes	19	37.3	16	31.4	0.391 / 0.53
	No	32	62.7	35	68.6	
Health insurance	Yes	48	94.1	45	88.2	1.097^a^ / 0.48
	No	3	5.9	6	11.8	
Family type	Nuclear	40	78.4	41	80.4	0.060 / 0.80
	Extended	11	21.6	10	19.6	
Perception of income status	Good	6	11.8	10	19.6	1.341 / 0.51
	Medium	37	72.5	35	68.6	
	Bad	8	15.7	6	11.8	
Total		51	100.0	51	100.0	

x^2^ : Chi-square test; a: Fisher’s exact test

X̄±Ss: Mean ± SD

Pregnant women who had never given birth before, had a single pregnancy, had reached at least 35 weeks of gestation, had no maternal contraindications that could affect breastfeeding (medication use, infectious diseases, psychiatric problems, etc.), and voluntarily participated in the study were included. Mothers who wished to withdraw from the study, those with newborn contraindications that could affect breastfeeding (presence of anomalies, stay in intensive care, etc.), those who could not be reached within the first five days after birth, and those who moved to another city/district were excluded from the study.

### Data collection tools

The data collection tools used during the study are as follows.:

- *Pregnant Introductory Form*: It was prepared by the researchers in line with the literature [12–14]. It consisted of 21 questions in total, including six items on sociodemographic characteristics, seven items on obstetric characteristics, and eight items on the opinions of pregnant women about breastfeeding.
- *Mother-Newborn Introduction Form*: It was prepared by the researchers in line with the literature and consisted of a total of 13 questions, including five items about the characteristics of the mother’s birth, three items about the characteristics of the baby, and five items about the first breastfeeding [12–14].
- *Breastfeeding Questionnaire*: It was prepared by the researchers in line with the literature [12, 13]. It consists of eight questions, including the characteristics of breastfeeding.
- *Breast Problems Diagnosis Form*: It was developed by the researchers in line with the literature for the detection of existing breast problems [14]. In the form, eight breast problems, including inward depression/flatness of the nipple, nipple pain, nipple crack, engorgement, duct obstruction, mastitis, breast abscess, and nipple fungus were examined.
- *LATCH Breastfeeding Diagnostic Measurement Tool*: Developed in 1993 by Deborah Jensen and Sheilla Wallace. The scale includes five evaluation criteria. For each criterion, 0,1,2 points are given. Breastfeeding success is evaluated by summing the scores. The highest score is 10, and the lowest score is 0. There is no cut-off point. The higher the scores obtained from the scale, the higher the breastfeeding success. The Turkish validity of the measurement tool was conducted by Yenal and Okumuş in 2003 and the Cronbach’s Alpha value was reported as 0.95 [15]. In this study, the Cronbach’s Alpha value of the scale was found to be 0.71.
- *Breastfeeding Self-Efficacy Scale (BSES)*: It is a 33-item scale developed by Cindy-Lee Dennis to measure breastfeeding competence. This 5-point Likert-type scale consists of two sub-dimensions: technical and personal thoughts. The higher the total score on the scale, the higher the breastfeeding competence. The lowest score is 33, and the highest score is 165. The Turkish validity and reliability of the scale were conducted by Ekşioğlu and Çeber in 2011. Cronbach’s Alpha value was reported as 0.91 [16]. In this study, the Cronbach’s Alpha value of the scale was found to be 0.94.

### Outcomes

The outcome variables of the study were exclusive breastfeeding of infants in the first six months postpartum, occurrence of breast problems, and breastfeeding competence of mothers.

### Intervention

This study was conducted in the form of breastfeeding support given using the wearable Lactation Simulation Model (LSM) in the antenatal period and follow-up and counseling on the fifth and fifteenth days, first, third, and sixth months in the postpartum period.

#### Antenatal period

Verbal and written consents were obtained from the pregnant women who agreed to participate in the study and met the inclusion criteria. It was emphasized that participants could withdraw from the study, which was based on voluntary participation, at any time. Contact information was collected from the pregnant women. Appointments were then scheduled for prenatal consultations. These consultations took place on a mutually agreed-upon date and time, in a room deemed suitable by the hospital management. Pregnant women in the intervention group completed a “Pregnant Women’s Information Form” and were given a “Breastfeeding and Breast Problems Education Booklet.” Individual breastfeeding training was provided using a LSM (Light Breast Simulation) and newborn model, supported by simulation. The LSM model is a wearable simulator that demonstrates breastfeeding positions, infant latching, breast milk expression, and nipple problems. The study used a show-and-tell technique to demonstrate correct breastfeeding techniques and positions, weaning, breast milk expression, and the identification of breast problems. Information on the composition and benefits of breast milk, initiation of breastfeeding, duration and frequency of breastfeeding, signs of breast milk adequacy, breast care, and breast problems and their solutions, also included in the provided booklet, was conveyed. Each pregnant woman’s training was completed in a single session, lasting approximately 65 min. The training was provided by a researcher with a midwifery background. The researcher holds a breastfeeding counseling certificate.

Pregnant women in the control group also completed a “Pregnancy Information Form” on the day they were included in the study. Additionally, they were given a “Breastfeeding and Breast Problems Education Booklet”.

#### Postpartum fifth, fifteenth day, and first month

Home visits were conducted by the same researcher who provided antenatal education to mothers on the fifth, fifteenth day, and first month after birth. Mothers in the intervention group received individual breastfeeding support in addition to completing the forms. Reminder information was repeated. Questions such as, “Does she give her baby any food other than breast milk?“, “Are there any breastfeeding problems?“, and “Are there any breast problems?” were asked. The mothers’ questions were answered. Mothers in the control group received no information about breastfeeding after completing the forms and were supported in participating in their postpartum follow-up and care at the Family Health Center. They were referred to the Family Health Center for any emerging breastfeeding problems and were registered there. *c)Postpartum third and sixth month*.

The study was conducted by the same researcher through telephone interviews with mothers at three and six months of age. Breastfeeding status and breast problems of the mothers in both groups were evaluated. “Does she give any food other than breast milk to her baby?” “Is there any problem with breastfeeding?” “Does she have breast problems?” were analyzed.

Reminder information was repeated with the mothers in the intervention group, and their questions were answered. Telephone counseling was continued throughout the follow-up. The researcher called the mother once a month. The mother could reach the researcher on weekdays during working hours. Mothers in the control group received no information about breastfeeding after completing the forms and were encouraged to participate in their ongoing follow-up and care at the Family Health Center. They were referred to the Family Health Center, where they were registered for breastfeeding problems that developed.

### Statistical analysis

Statistical Package for the Social Sciences (SPSS v. 15.0, Chicago, IL, USA) was used to evaluate the data. The suitability of the data for normal distribution was evaluated by the Shapiro-Wilk test. As a result of the test, it was determined that the data did not show normal distribution. Descriptive statistics, numbers, percentages for categorical data, and mean, median, and interquartile ranges for continuous data were shown. The chi-square test and Mann-Whitney U test were used for intergroup comparisons, and the Friedman test was used for intra-group comparisons.

## Results

Table 1 presents the sociodemographic characteristics of the pregnant women who participated in the study. The mean age was 25.44 ± 3.37 years. It was observed that 43.1% of the pregnant women in the intervention group were between the ages of 25–29, 54.9% were university graduates, 62.7% were not working, and 94.1% had health insurance. It was determined that 78.4% of the pregnant women had a nuclear family, and 72.5% had a medium-perceived income. It was determined that 49% of the control group was in the age group of 20–24 years, and 51% had an educational level of high school and below. It was found that 68.6% of the pregnant women were not working, and 88.2% had health insurance. It was observed that 80.4% of the pregnant women had a nuclear family structure. The proportion of pregnant women who defined their income status as medium was 68.6% (Table 1).

Table 2 presents the feeding patterns and breastfeeding characteristics of the babies in the first six months postpartum. It was observed that 96.1% of the mothers in the intervention group fed their babies with breast milk on the fifth day and 92.2% on the sixth month (exclusive breastfeeding, expressing breast milk, or formula-assisted breastfeeding). When duration of a breastfeeding session was analyzed, it was found that a duration of a breastfeeding session 10–20 min in the first three follow-ups (50.0% on the 5th day, 57.1% on the 15th day, 55.1% in the first month), and these rates decreased to 33.3% and 19.1% in the third and sixth-month follow-ups, respectively. While the rate of bottle use in this group was 31.4% on the fifth day, this rate was 27.5% in the sixth month. In the control group, 90.2% of the mothers continued to feed their babies with breast milk (exclusive breastfeeding, expressing breast milk, or formula-assisted breastfeeding) on the fifth day and 88.2% on the sixth month.

**Table 2:** Feeding patterns and breastfeeding characteristics of intervention and control group mothers for their babies in the first six months after birth

Characteristics	Intervention group (*n* = 51)					Control group (*n* = 51)				
	5.day *n*(%)	15.day *n*(%)	1.month *n*(%)	3.month *n*(%)	6.month *n*(%)	5.day *n*(%)	15.day *n*(%)	1.month *n*(%)	3.month *n*(%)	6.month *n*(%)
Baby’s feeding style										
Feeding with breast milk	49 (96.1)	50 (98.0)	49 (96.1)	48 (94.1)	47 (92.2)	45 (88.2)	46 (90.2)	46 (90.2)	46 (90.2)	45 (88.2)
Formula feeding	2 (3.9)	1 (2.0)	2 (3.9)	3 (5.9)	4 (7.8)	6 (11.8)	5 (9.8)	5 (9.8)	5 (9.8)	6 (11.8)
Breastfeeding frequency *										
Whenever the baby wants	27 (61.4)	25 (51.0)	26 (53.1)	20 (41.7)	34 (72.3)	29 (65.9)	25 (54.4)	26 (56.5)	28 (60.9)	29 (64.4)
2 h	11 (25.0)	16 (32.7)	11 (22.4)	6 (12.5)	2 (4.3)	4 (9.1)	10 (21.7)	5 (10.9)	5 (10.9)	-
3 h and above	6 (13.6)	8 (16.3)	12 (24.5)	22 (45.8)	11 (23.4)	11 (25.0)	11 (23.9)	15 (32.6)	13 (28.2)	16 (35.6)
Duration of a breastfeeding session (min)*										
As much as the baby wants	6 (13.6)	7 (14.3)	6 (12.2)	13 (27.1)	15 (31.9)	10 (22.7)	10 (21.7)	13 (28.3)	14 (30.4)	29 (64.4)
5–10 min	11 (25.0)	11 (22.4)	15 (30.6)	19 (39.6)	23 (48.9)	21 (47.7)	19 (41.3)	23 (50.0)	17 (37.0)	11 (24.4)
10–20 min	22 (50.0)	28 (57.1)	27 (55.1)	16 (33.3)	9 (19.1)	4 (9.1)	12 (26.1)	8 (17.4)	14 (30.4)	5 (11.2)
20–30 min	5 (11.4)	3 (6.1)	1 (2.0)	-	-	9 (20.5)	5 (10.9)	2 (4.3)	1 (2.2)	-
Reasons for not breastfeeding **										
No breast milk	1 (14.2)	-	2 (100.0)	3 (100.0)	4 (100.0)	2 (28.6)	4 (80.0)	5 (100.0)	5 (100.0)	6 (100.0)
Breast rejection	3 (42.9)	-	-	-	-	1 (14.3)	1 (20.0)	-	-	-
No nipples	3 (42.9)	2 (100.0)	-	-	-	4 (57.1)	-	-	-	-
Using a bottle										
Yes	16 (31.4)	15 (29.4)	12 (23.5)	12 (23.5)	14 (27.5)	31 (60.8)	30 (58.8)	33 (64.7)	37 (72.5)	41 (80.4)
No	35 (68.6)	36 (70.6)	39 (76.5)	39 (76.5)	37 (72.5)	20 (39.2)	21 (41.2)	18 (35.3)	14 (27.5)	10 (19.6)
Using a pacifier										
Yes	27 (52.9)	30 (58.8)	29 (56.9)	28 (54.9)	27 (52.9)	27 (52.9)	35 (68.6)	36 (70.6)	37 (72.5)	37 (72.5)
No	24 (47.1)	21 (41.2)	22 (43.1)	23 (45.1)	24 (47.1)	24 (47.1)	16 (31.4)	15 (29.4)	14 (27.5)	14 (27.5)
Time to switch to complementary feeding										
6. months	-	-	-	-	36 (70.6)	-	-	-	-	22 (43.2)
Between 4–6 months	-	-	-	-	15 (29.4)	-	-	-	-	29 (56.8)

* Only breastfeeding mothers were included

** It was evaluated on mothers who expressed their babies and fed them with breast milk and/or only formula

Regarding the duration of a breastfeeding session, 47.7% on the fifth day, 41.3% on the 15th day, 50.0% on the first month, 37.0% on the third month, and 24.4% on the sixth month breastfed for 5–10 min. The rates of bottle use were 60.8% on day five and 80.4% on month six. It was determined that 70.6% of the mothers in the intervention group and 43.2% of the mothers in the control group switched to complementary feeding at the appropriate time/ sixth month (Table 2).

Table 3 shows the rates of exclusive breastfeeding of mothers. In the intervention group, 64.7% of the mothers on the fifth day, 66.7% on the fifteenth day, 76.5% on the first month, 76.5% in the third month, and 54.9% in the sixth month fed their babies exclusively with breast milk. In the control group, 35.3% were on the fifth day, 35.3% were on the fifteenth day, 33.4% were on the first month, 23.5% were on the third month, and 21.6% were on the sixth month. A statistically significant difference was found between the intervention and control groups on the fifth day, fifteenth day, first month, third month, and sixth month in terms of exclusive breastfeeding status (*p* < 0.001, Table 3).

**Table 3:** The status of intervention and control group mothers in feeding their babies exclusive breastfeeding of mothers in the first six months after birth

Characteristics		Intervention group		Control group		Significance test
		*n*	%	*n*	%	x^2^ / *p*
5.day	Yes	33	64.7	18	35.3	8.824 / 0.00
	No	18	35.3	33	64.7	
15.day	Yes	34	66.7	18	35.3	10.043 / 0.00
	No	17	33.3	33	64.7	
1.month	Yes	39	76.5	17	33.4	19.165 / 0.00
	No	12	23.5	34	66.7	
3.month	Yes	39	76.5	12	23.5	28.588 / 0.00
	No	12	23.5	39	76.5	
6.month	Yes	28	54.9	11	21.6	10.674 / 0.00
	No	37	45.1	40	78.4	
Total		51	100.0	51	100.0	

x2 : Chi-square test

Table 4 shows the breast problems experienced by the mothers included in the study during the first six months after delivery. It was determined that mothers most frequently experienced nipple pain in the first days after delivery. The rate of nipple pain was 35.3% on the 5th and 15th postpartum days in the intervention group and 58.8% and 51.0% in the control group, respectively. There was a significant difference between the intervention and control groups in terms of nipple pain on the fifth day and first month (*p* < 0.05). Nipple cracking was also seen most frequently on the 5th and 15th days. The rates were 23.5% and 17.6% in the intervention group on day five and day 15, respectively, and 45.1% and 41.2% in the control group, respectively. In all follow-ups, nipple cracks were more common in the intervention group compared to the control group. On the fifth and fifteenth days, a significant difference was found in terms of nipple cracking in the intervention and control groups (*p* < 0.05). Engorgement was observed most frequently on the fifteenth day, with a rate of 15.7% in the intervention group, and most frequently on the fifth day, with a rate of 31.4% in the control group. Engorgement was observed more frequently in all follow-ups in mothers in the control group. A significant difference was found between the mothers in the intervention and control groups on the fifth day and in the first month (*p* < 0.05). It was determined that the problem of blocked ducts was observed most frequently on the fifteenth day in both groups, and the rates were 3.9% in the intervention group and 19.6% in the control group. On day 15, a significant difference was found between the mothers in the intervention and control groups in terms of the problem of blocked ducts (*p* < 0.05, Table 4).

**Table 4:** Breast problems experienced by mothers in the intervention and control groups in the first six months after birth*

Characteristics		Intervention group		Control group		Significance test
		*n*	%	*n*	%	x^2^ / *p*
Nipple pain	5.day	18	35.3	30	58.8	5.667 / 0.01
	15.day	18	35.3	26	51.0	2.558 / 0.11
	1.month	9	17.6	24	47.1	10.079 / 0.00
	3.month	4	7.8	11	21.6	3.830^a^ / 0.09
	6. month	12	23.5	14	27.5	0.206 / 0.65
Nipple cracking	5.day	12	23.5	23	45.1	5.263 / 0.02
	15.day	9	17.6	21	41.2	6.800 / 0.00
	1.month	3	5.9	7	13.7	1.774 / 0.31
	3.month	-	-	1	2.0	-
	6.month	-	-	-	-	-
Engorgement	5.day	5	9.8	16	31.4	7.256 / 0.00
	15.day	8	15.7	14	27.5	2.086^a^ / 0.22
	1.month	6	11.8	14	27.5	3.980 / 0.04
	3.month	-	-	3	5.9	-
	6. month	-	-	-	-	-
Blocked ducts	5.day	-	-	3	5.9	-
	15.day	2	3.9	10	19.6	6.044^a^ / 0.02
	1.month	1	2.0	4	7.8	1.893^a^ / 0.36
	3.month	-	-	1	2.0	-
	6.month	-	-	-	-	-
Total		51	100.0	51	100.0	

x2 : Chi-square test

a: Fisher’s exact test

* During the follow-up of the study, in addition to the breast problems listed in the table above, mastitis, breast abscess, and candida in the nipple were questioned, but since no cases were encountered, they were not included in the table

** Empty cells were not analyzed due to insufficient cell count

When the total scores of the LATCH Breastfeeding Diagnostic Measurement Tool were evaluated, it was determined that there was a difference between the scale scores of the intervention and control groups when the fifth, fifteenth, and first-month follow-ups were compared (*p* < 0.05). When the intervention and control groups were compared, it was found that the total scale score of the intervention group was different from the control group on the fifth and fifteenth days (*p* < 0.05). According to the results of the comparison between the groups in terms of the mean scores of BSES, Personal Thoughts Subscale, and Technical Subscale, it was found that the mean scale score of the intervention group was different from the control group on the fifth and fifteenth days and at the first month (*p* < 0.05, Table 5).

**Table 5:** Comparison of LATCH Diagnostic Measurement Tool, BSES total and subscale scores of mothers in the intervention and control groups

Characteristics		Intervention group (*n* = 51)			Control group ( *n* = 51)			Significance test	Effect size
		n*	Mean Rank	Median (IQR)	n*	Mean Rank	Median (IQR)	z / *p*	
LATCH Diagnostic Measurement Tool Total Score	5.day	44	59.55	8.00 (2.00)	44	29.45	5.50 (2.00)	**-5.608 / 0.00**	-1.473
	15.day	49	55.80	9.00 (2.50)	46	39.70	8.00 (1.25)	**-2.919 / 0.00**	-0.661
	1.month	49	50.62	9.00 (2.00)	46	45.21	9.00 (1.00)	-1.020 / 0.30	-0.230
		**x**^**2**^: **29.450 / p: 0.00**			**x**^**2**^: **69.298 / p: 0.00**				
BSES Total Scale Score	5.day	44	56.86	141.00 (25.25)	44	32.14	125.00 (17.00)	**-4.542 / 0.00**	-1.060
	15.day	49	59.46	144.50 (13.75)	46	35.79	132.00 (19.50)	**-4.184 / 0.00**	-0.871
	1.month	49	58.99	152.00 (9.00)	46	36.29	140.50 (24.25)	**-4.013 / 0.00**	-0.919
		**x**^**2**^: **23.902 / p: 0.00**			**x**^**2**^: **30.655 / p: 0.00**				
BSES Personal Thoughts Subscale	5.day	44	51.40	83.00 (17.00)	44	37.60	75.00 (17.25)	**-2.535 / 0.01**	-0.587
	15.day	49	56.14	84.00 (11.50)	46	39.33	75.50 (13.75)	**-2.974 / 0.00**	-0.636
	1.month	49	56.36	87.00 (7.75)	46	39.10	82.00 (20.75)	**-3.055 / 0.00**	-0.768
		**x**^**2**^: **16.551 / p: 0.00**			**x**^**2**^: **10.024 / p: 0.00**				
BSES Technical Subscale	5.day	44	57.81	59.00 (13.00)	44	31.19	49.00 (7.00)	**-4.892 / 0.00**	-1.044
	15.day	49	59.45	62.00 (7.00)	46	35.80	55.00 (8.00)	**-4.186 / 0.00**	-0,908
	1.month	49	58.24	66.00 (4.00)	46	37.09	61.00 (14.50)	**-3.750 / 0.00**	-0.843
		**x**^**2**^: **16.051 / p: 0.00**			**x**^**2**^: **34.714 / p: 0.00**				

*z*: Mann Whitney *U* Test; x^2^: Friedman Test

* Breastfeeding mothers were evaluated

Statistically significant results are shown in bold

## Discussion

The gold standard of global public health is considered to be babies who are exclusively breastfed for the first six months [17]. In the report published by WHO and UNICEF in 2019, it is stated that it is aimed to feed at least 70% of infants younger than six months exclusively with breast milk [5, 18]. However, when the literature is examined, it is reported that exclusive breastfeeding rates in the first month after birth vary between 68% and 84%, and exclusive breastfeeding rates during the first six months are between 26.5% and 48.6% [19–21]. The rates in Türkiye are similar [6, 22]. This is an indication that mothers need support in the breastfeeding process.

In this study, exclusive breastfeeding rates remained at a certain rate in the intervention group (64.7%-54.9%) and decreased in the control group (35.3%-21.6%) from the fifth to the sixth month of follow-up, respectively. When randomized controlled studies in which breastfeeding education and support were provided were examined, it was observed that the rates of exclusive breastfeeding for the first six months varied between 67.8% and 26.5% in the intervention groups and between 28.0% and 3.3% in the control groups [22, 23]. As a result of Ekşioğlu’s (2017) study in which she provided breastfeeding training in the postnatal period using a simple simulator, she reported that the rate of exclusive breastfeeding of the mothers in the intervention group was 88.3% in the second week of postpartum follow-up and 56.6% in the sixth-month follow-up. It can be said that the research data are similar to those of our study. According to the TDHS 2018 report, the rate of exclusive breastfeeding for the first six months in Türkiye was 41.0%. The difference in the rates of exclusive breastfeeding in the first six months in the studies is thought to be due to the years of study, the evaluation period of the exclusive breastfeeding variable, and differences in training, support, and follow-up techniques. All of the studies mentioned above show that mothers who received breastfeeding education had higher rates of exclusively breastfeeding for the first six months compared to control groups, indicating that the education was effective.

It is known that initiation and maintenance of breastfeeding in the postpartum period is associated with mothers’ adaptation to the puerperium, postpartum pain, and other problems [24]. The most common reason for mothers to experience difficulties in the breastfeeding period is breast problems that frequently occur in the first two weeks [25]. Breast problems experienced, especially in this period, cause the mother to define breastfeeding as a painful situation and to develop a negative attitude towards breastfeeding [26]. The most common breast problems experienced by mothers have been reported as painful nipples and/or cracks, engorgement, blocked duct, and mastitis [27, 28]. In this study, it was found that nipple pain, nipple crack, and mastitis were observed with rates of 35.3%, 23.5%, and 9.8%, respectively, in the intervention group on the fifth day, whereas they were observed with rates of 58.8%, 45.1%, and 31.4%, respectively, in the control group (Table 4).

Nipple pain frequently occurs in the first 48 to one week after delivery, with or without visible damage [29]. In the literature, nipple pain was reported with a rate of 57.6% on the first postpartum day and 42.4% on the fourth postpartum day [30]. The rates of nipple pain on the fifth day in the control group were similar. Nipple pain experienced in the first postpartum days is generally associated with incorrect breastfeeding position and latching [31]. International organizations recommend that mothers should be given breastfeeding training by a professional lactation consultant before starting breastfeeding and that their incorrect grasping and positions should be corrected. In this study, it was expected that the rate of experiencing nipple pain would be low because the breastfeeding education/support given to the mothers in the intervention group included breastfeeding positions and correct grasping techniques.

The rates of nipple cracking continued to decrease in both groups throughout the six-month follow-up. A significant difference was found between the two groups in terms of nipple cracking on the fifth and fifteenth days (*p* < 0.05, Table 4). In a study, the effect of different breastfeeding education techniques on nipple cracking was examined, and it was reported that the rates of nipple cracking in the second postpartum week were 63.3% in the group receiving routine care, 56.7% in the group receiving brochures and 20.0% in the group receiving demonstration-based education. At the end of the fourth week, this rate was reported to be 30.0% in the group receiving routine care and less than 10% in the other two groups [14]. It can be said that the findings of the study are similar. The content of breastfeeding support given to the intervention group in the antenatal period must include precautions and solution suggestions for breast problems. At the same time, it is thought that teaching breastfeeding positions, which are the most responsible for the formation of nipple cracks, by demonstrating them on the simulator with the show-and-do technique and following the breastfeeding process with follow-up counseling affected the incidence of nipple cracks in the intervention group less than in the control group.

In the study, a difference in the occurrence of engorgement was observed between the two groups at the fifth day and first month follow-up (*p* < 0.05, Table 4). It is known in the literature that two-thirds of mothers experience breast engorgement at some point after delivery [32, 33]. Even if a mother applies correct breastfeeding techniques, engorgement may occur due to some physiological reasons. This information explains the increase in the intervention group on the fifteenth day (9.8% on the fifth day and 15.7% on the fifteenth day). At this point, mothers need to know the techniques to cope with engorgement so that engorgement does not turn into a problem of blocked duct. Hence, although the rate of blocked duct was low in both groups, it was observed that the rate of blocked duct on the fifteenth day made a difference between the two groups (*p* < 0.05, Table 4).

Mastitis, breast abscess, and candida infection were not observed during all follow-ups. In the literature, it is observed that these problems occur more frequently in studies planned as follow-up studies [34]. This may be associated with home visit follow-up during the first month postpartum. Although no intervention was made in the control group during the home visit, the fact that high-risk breast problems were not experienced in the control group may be explained by the fact that the training booklet given during the antenatal period raised awareness. The mothers who were seen to have breast problems during the home visit were referred to the family health center in the early period. Providing professional support to the woman at home by the midwife during this period led to a lower incidence of all breast problems in the intervention group.

In the study, the breastfeeding competencies of mothers were evaluated with breastfeeding success and self-efficacy. Success and self-efficacy are factors that increase or decrease each other. They are also among the important factors affecting the duration of breastfeeding and the duration of exclusive breastfeeding in the postpartum period [11]. In this study, breastfeeding success was evaluated on the fifth day, fifteenth day, and first month postpartum. It was found to be 7.79 ± 1.39 at the first follow-up and 9.02 ± 0.87 at the last follow-up in the intervention group and 5.72 ± 1.42 at the first follow-up and 8.82 ± 0.87 at the last follow-up in the control group. The results showed that mothers in the intervention group had higher breastfeeding success scores than the control group in all follow-ups (Table 5). When studies in the literature similar to this study design, in which breastfeeding support was provided using a simulator in the antenatal period and breastfeeding success was evaluated, it is seen that the results are similar and supportive of this study [13, 14]. In addition, breastfeeding success scores were found to increase in both groups in this study (Table 5). It was expected that the breastfeeding success score would be higher in the intervention group receiving antenatal breastfeeding education compared to the control group. The increase in the score in the control group may be explained by the strengthening of the baby’s sucking power and the mother’s gaining experience and discovering the position in which she can breastfeed without pain [35].

The breastfeeding self-efficacy of mothers is an important factor contributing to positive breastfeeding outcomes [11]. Each increase in breastfeeding self-efficacy score means that the probability of the mother to continue breastfeeding is 4.0%-7.0% higher [36]. As a result of a study, it was reported that low self-efficacy resulted in a perception of inadequate breast milk, which was associated with formula use [37]. In this study, the mean self-efficacy score of the mothers in the intervention group was 141.61 ± 18.24 at the first follow-up on the fifth postpartum day and 151.46 ± 8.8 at the last follow-up in the first month. In the control group, the mean scores were 123.93 ± 14.95 at the first follow-up and 137.23 ± 20.28 at the last follow-up (Table 5). Arslan (2020) provided breastfeeding support in the antenatal period using the LSM used in this study and found that the postpartum self-efficacy of the group that did not use the simulator was lower. In different studies comparing self-efficacy after breastfeeding training, it was found that the scores of the mothers in the intervention group were higher than those in the control group [12, 38, 39]. The reason for the similar results of the studies is thought to be antenatal education followed by postnatal follow-up. At this point, it is important to keep the mother’s self-efficacy high in the period when she has to manage the breastfeeding process alone after discharge. Midwives must provide professional support to increase women’s breastfeeding self-efficacy.

## Conclusions

The rate of exclusive breastfeeding for the first six months was significantly higher in the intervention group compared to the control group. In postnatal follow-ups, it was determined that the intervention group experienced breast problems less frequently compared to the control group. It was found that mothers in the intervention group breastfed more successfully and had higher breastfeeding self-efficacy than the control group. Accordingly, it may be recommended that a supportive care model be established in the management of postpartum care.

### Limitation

This study was conducted on pregnant women admitted to a hospital. Therefore, the results obtained from this study are valid only for pregnant women in the province where the study was conducted and cannot be generalized to all pregnant women.

## Data Availability

The datasets generated and/or analysed during the current study are not publicly available but are available from the corresponding author on reasonable request.
